# Protein kinase C theta: evolution, regulation, and function

**DOI:** 10.1042/BCJ20250280

**Published:** 2026-04-13

**Authors:** Stefanie J. Hodapp, Gerard Manning, Alexandra C. Newton

**Affiliations:** 1 Department of Pharmacology, University of California, San Diego, La Jolla, CA 92037, U.S.A.; 2 Biomedical Sciences Graduate Program, University of California, San Diego, La Jolla, CA 92037, U.S.A.; 3 Nuabio Research, Burlingame, CA 94010, U.S.A.

**Keywords:** immunology, protein kinase C, signalling

## Abstract

Protein kinase C (PKC) is a family of serine/threonine kinases whose nine members play central roles in signal transduction, regulating key cellular processes such as proliferation, migration, differentiation, and apoptosis. PKC theta (θ) is a member of the diacylglycerol-regulated novel-d PKC subfamily and differentiates itself from other family members in its selective expression in hematopoietic cells, where it is essential for T cell activation, differentiation, and survival. Given its pivotal role in immune signaling, dysregulated PKCθ has been implicated in inflammatory and autoimmune diseases, as well as cancer. However, unlike the extensively studied Ca^2+^-regulated conventional PKC isozymes, the mechanisms of PKCθ and its roles in both health and disease remain relatively unexplored. In this review, we examine the evolution of PKCθ, its domain structure and lifecycle, its role in immune signaling, and its relevance in disease.

## Introduction

The serine/threonine kinase, protein kinase C (PKC), is a key regulator of signal transduction, controlling diverse cellular processes such as proliferation, differentiation, and apoptosis [1]. Among the nine PKC isozymes, PKC theta (θ) (human gene *PRKCQ*) is gaining increasing interest because of its role in immune signaling. Uniquely, PKCθ is predominantly expressed in hematopoietic cells, particularly in T lymphocytes, where it mediates T cell receptor (TCR) signaling to regulate the immune response [2]. Upon TCR engagement, PKCθ is recruited to the immunological synapse, where it initiates a cascade of signals leading to the activation of transcription factors NF-κB, AP-1, and NFAT [3–7]. These transcription factors drive the expression of genes involved in T cell activation, survival, and differentiation [8]. With its unique localization and function, PKCθ serves as a crucial checkpoint in immune signaling. Dysregulation of PKCθ has been implicated in a variety of diseases, including autoimmune and inflammatory disorders, as well as cancer [9–11]. Emerging evidence suggests that targeting PKCθ could modulate immune responses in a disease-specific manner, positioning it as a promising therapeutic target. This review explores the evolution of the PKC family, the structure and function of PKCθ, and its implications in various pathological conditions.

## PKC evolution

The PKC family is encoded by nine genes originally grouped into three subfamilies: conventional (cPKC; α, βI/II, γ), novel (nPKC; δ, ε, η, θ), and atypical (aPKC; ζ, ι) [12,13]. Family members were initially classified based on second messenger requirements of the encoded proteins, as determined by biochemical assays [12]. The cPKCs require both diacylglycerol (DG) and Ca^2+^ for activation, whereas the nPKCs are activated by DG alone [12], and the aPKCs are activated by the lipid second messenger sphingosine-1-phosphate [14] or by binding to protein scaffolds [12]. Additionally, the activity of all isozymes is stimulated by highly cooperative binding to phosphatidylserine [15,16]. A defining feature of all family members is that binding interactions of the regulatory modules unleash catalytic activity, with subfamilies defined by what these binding interactions are.

**Figure 1:**
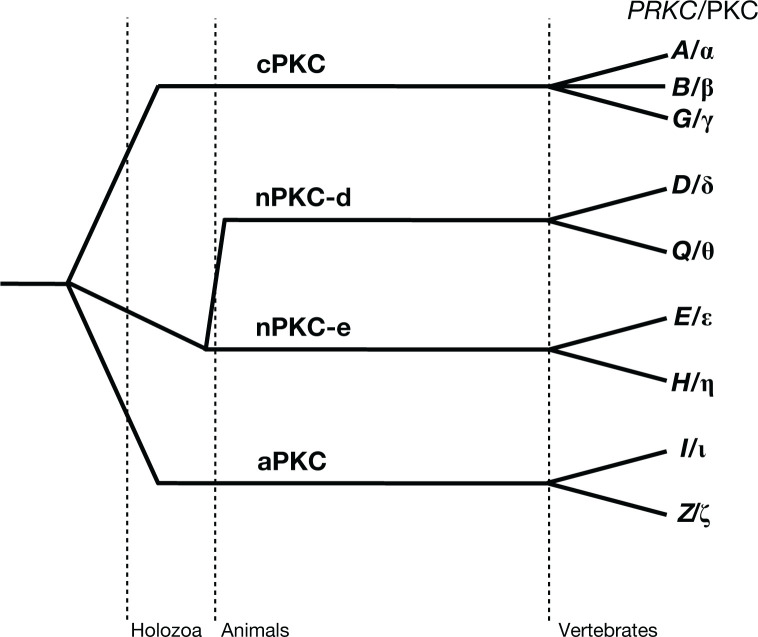
Evolution of the PKC family PKC isozymes are grouped into three main subclasses: conventional (cPKC; α, βI/II, γ), novel (nPKC; δ, ε, η, θ), and atypical (aPKC; ζ, ι); gene names of *PRKC* family members indicated in italics on right. Evolutionary analyses revealed that the novel subclass evolved into two distinct subfamilies distinguishing PKCδ and PKCθ from PKCε and PKCη, referred to here as novel-d and novel-e PKC subfamilies, respectively.

**Figure 2:**
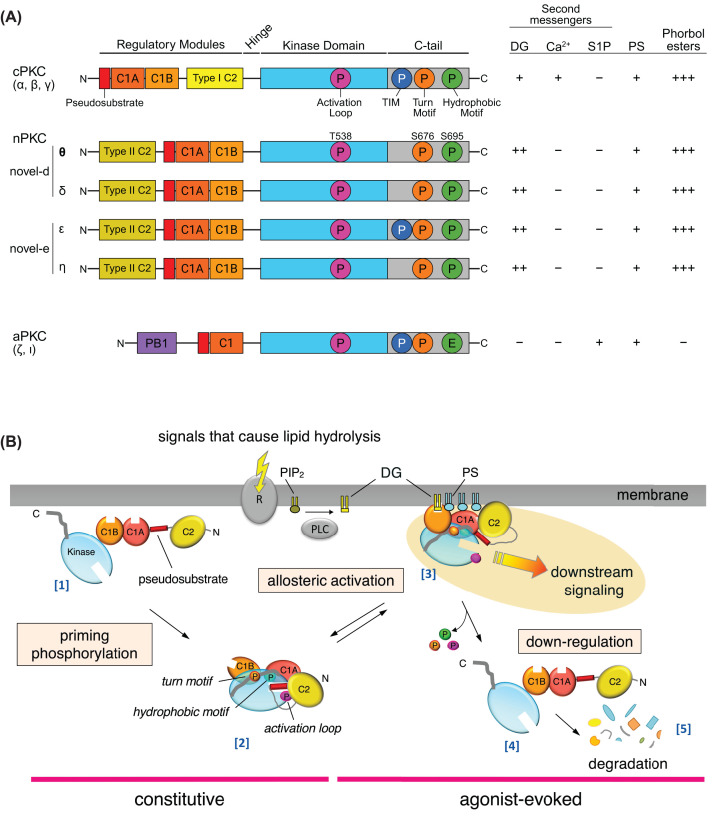
Primary structure and lifecycle of PKC (**A**) The nine PKC isozymes are grouped into four subclasses, conventional (cPKC), novel-d and novel-e (nPKC), and atypical (aPKC), based on primary structure (left) and second messenger requirements (right). All isozymes contain an autoinhibitory pseudosubstrate (red) followed by a C1A domain (dark orange), kinase domain (cyan), and C-terminal tail (gray). cPKCs and nPKCs contain an additional DG-sensing C1B domain (light orange) and a Ca^2+^-sensing Type I (yellow) or Ca^2+^-insensitive Type II C2 domain (dark yellow). aPKCs have a PB1 domain (purple) that mediates binding to protein scaffolds. Three conserved priming phosphorylations are indicated: activation loop (pink; T538 in PKCθ), turn motif (orange; S676 in PKCθ), and hydrophobic motif (green; S695 in PKCθ); aPKCs have a Glu at the hydrophobic motif. The mTORC2-regulated kinases, the cPKCs, the aPKCs, and PKCε, have additional TIM phosphorylation site (blue). DG, diacylglycerol; PS, phosphatidylserine; S1P, sphingosine-1-phosphate. (**B**) Cartoon of lifecycle using nPKC as an example. Newly synthesized PKC is in an open and unprimed state that is susceptible to degradation (i). Following phosphorylation at the activation loop (by PDK1 for all isozymes), turn motif (either mTORC2 for kinases with TIM site or autophosphorylation for PKCθ), and hydrophobic motif (autophosphorylation for all isozymes with this site), PKC adopts an autoinhibited conformation in which the pseudosubstrate occupies the substrate-binding pocket of the kinase domain. The C2 domain clamps over the pseudosubstrate holding it in place to prevent substrate binding (ii). Receptor-mediated hydrolysis of PIP_2_ to generate DG (yellow lipid) results in recruitment of PKC to cell membranes, where PKC cooperatively binds PS (blue lipid), inducing a reversible conformational change that releases the pseudosubstrate from the substrate-binding pocket, enabling downstream signaling (iii). Binding to protein scaffolds can fine-tune the membrane localization of PKC isozymes (not shown). PKC in the open/active conformation is sensitive to dephosphorylation (iv) and subsequent proteasome-mediated degradation. As a result, prolonged activation, as occurs with phorbol esters, results in down-regulation (v).

Although the novel subclass was historically considered a single subfamily [17], Manning and colleagues first noted that novel PKC isozymes comprise two distinct subfamilies based on evolutionary conservation [18]. Specifically, PKCθ and delta (δ) belong to a different subfamily from PKC epsilon (ε) and eta (η) [18]. Henceforth, we refer to these as novel-d and novel-e PKC subfamilies, respectively, thus expanding to four PKC subfamilies (Figure 1). Evolutionary analysis indicates that the conventional, atypical, and novel-e families emerged in holozoa, whereas the novel-d family is only in animals (Figure 1). The novel-d and novel-e retain the same domain composition and order of domains (Figure 2A) but differ in conserved subfamily-specific residues. Notably, the C2 domain harbors several differences in amino acids predicted to be at domain interfaces. For example, a conserved LKPT motif in novel-e (residues 18–21 in PKCε), predicted to interface with the C-tail, is not conserved in novel-d, and a charge switch from D (D47 in PKCε) to K (K34 in PKCθ) is predicted to interface with the C1B. Additionally, QR in the linker between the C1A and C1B (Q235 in PKCε) is ER in novel-d (E224 in PKCθ), and EE in the C-tail (E723 in PKCε) is poorly conserved in novel-d PKC. The predicted positions of these differences between, but not within, the subfamilies suggest that they may uniquely control the autoinhibited conformation or activation dynamics of the subfamily members. Consistent with this, live cell imaging experiments have revealed that the novel-d isozymes are effectively autoinhibited, whereas the novel-e isozymes are weakly autoinhibited: PKCθ and PKCδ have almost no basal activity in cells [11,19], whereas PKCε and PKCη have high basal activity [19,20]. Thus, novel-d isozymes are under tight second messenger regulation, whereas the novel-e isozymes signal in the absence of second messenger stimulation, likely arising from differences in residues involved in maintaining the autoinhibited conformation.

## PKCθ domain composition

All PKCs share a conserved structure consisting of an N-terminal regulatory moiety that allosterically regulates a C-terminal kinase domain, followed by a C-terminal tail that serves as a docking site for regulatory proteins (Figure 2A) [21]. The regulatory moiety contains an autoinhibitory module comprising the pseudosubstrate segment, which binds the substrate-binding cavity, linked to a C1A domain, which functions together to maintain the enzymes in an “off” state. cPKCs as well as novel-d and novel-e PKCs have a second C1 domain (C1B) that serves as the DG sensor. cPKCs are further distinguished by a Ca^2+^-sensing C2 domain that binds phosphatidylinositol 4,5-bisphosphate, recruiting cPKCs to the plasma membrane in response to elevations in intracellular Ca^2+^ [12,22]; novel-d and novel-e PKCs are insensitive to Ca^2+^ due to a novel C2 domain that lacks the acidic residues required for Ca^2+^ coordination that are present in the C2 of cPKCs [23]. Notably, novel-d and novel-e PKCs bind DG with two orders of magnitude higher affinity than cPKCs, eliminating the need for Ca^2+^ for membrane recruitment [24]. In contrast, aPKCs lack a C2 domain and are thus Ca^2+^-insensitive, and contain only one C1 domain; a basic rim surrounding the binding pocket of this C1 domain renders aPKCs insensitive to DG, although phosphatidylserine recognition is retained as in other isozymes [12]. Although the isolated C1A domains bind ligands, the ligand-binding pocket is masked in the context of the mature full-length protein. Thus, for PKCθ, the C1B domain, and not the C1A, is the primary binder of DG [25]. Unique to PKCθ, a Pro-rich targeting motif in the otherwise deeply conserved hinge separating the regulatory and kinase moieties controls its localization in T cells [26]. In these cells, engagement of the TCR and co-receptor CD28 results in recruitment of PKCθ to the immunological synapse [26].

## PKCθ expression

Transcriptomic analyses [27,28] reveal high expression of PKCθ in T cells and natural killer cells [27], as well as lower expression in innate lymphoid cells, mucosal-associated invariant T cells, microglia, common lymphoid progenitors, and several myeloid populations (immgen.org) [29]. Notably, other hematopoietic cells, including neutrophils, B cells, monocytes, and macrophages, do not express PKCθ [30]. In addition to these immune cells, PKCθ expression is also enhanced in the brain in oligodendrocytes, oligodendrocyte precursor cells, and astrocytes (proteinatlas.org) [27]. Lastly, its expression is enhanced in skeletal myocytes and platelets, and to a lesser extent in neurons. Although mRNA expression does not correlate with protein expression for most PKC isozymes, numerous studies have established PKCθ protein is abundant in immune cells [31–34] and skeletal muscle [35], and is also detected in neurons [36,37].

## Processing versus agonist-stimulated phosphorylation of PKCθ

All PKC isozymes are regulated by three ordered and constitutive phosphorylation events at the activation loop, turn motif, and hydrophobic motif, which correspond to Thr538, Ser676, and Ser695 in PKCθ, respectively (Figure 2A; pink, orange, and green circles) [38–40]. For the more extensively characterized cPKCs, newly synthesized PKC is first phosphorylated by mTORC2 at a recently identified threonine site termed the TOR-interaction motif (TIM) [41] and the turn motif to facilitate subsequent phosphorylation of the activation loop by the upstream kinase PDK1, in turn triggering autophosphorylation at the hydrophobic motif [42]. PKCθ is not regulated by mTORC2 and does not have the newly identified TIM. Unlike most PKC isozymes, *E. coli*-expressed kinase domain of PKCθ is phosphorylated at the turn motif, activation loop, and hydrophobic motif, revealing that all three sites are able to be modified by autophosphorylation [43,44]. However, studies in Jurkat and primary T cells establish a clear dependence on PDK1 for phosphorylation of the activation loop [45,46], although one report suggests that the Ser/Thr kinase MAP4K3 (also referred to as KHS2 or GLK) may also modify the activation loop in cells [47]. We note that substrate-motif analysis ranks PDK1 as the top kinase predicted to phosphorylate the activation loop, with MAP4K3 ranked 30th [48], and further studies are needed to clarify specific contexts where PDK1 may not be the major kinase modifying the site. Phosphorylation of the activation loop in T cells is induced by anti-CD3 or interferon stimulation [45,49]. Similar to cPKCs, the hydrophobic motif is regulated by autophosphorylation [43,44], with a recent mechanistic analysis showing conversion to a pseudokinase that maintains the conformation of an active, ATP-bound kinase domain prevents phosphate incorporation at this site [50]. Biochemical studies reveal that phosphorylation of the activation loop is necessary for PKCθ catalytic activity, whereas phosphorylation of the turn motif and hydrophobic motif fine-tunes its catalytic activity [43,51,52].

Once phosphorylated at the activation loop, turn motif, and hydrophobic motif, PKC adopts an autoinhibited conformation in which the pseudosubstrate binds the active site to prevent kinase activity (Figure 2B; [2]) [53–55]. In this state, PKC is inactive but poised to respond to second messengers. Importantly, any PKC that is unable to properly autoinhibit, such as variants with mutations that disrupt interdomain interactions, is subjected to degradation [56,57].

In addition to the processing phosphorylations, agonist-stimulated phosphorylations on PKCθ have been described and are likely to play important roles in tuning activity, localization, or stability. Notable are the phosphorylation of Y90 in the C2 domain and T219 in the linker between the two C1 domains [40]. Y90 is only conserved in mammals, suggesting a fast-evolving function; its phosphorylation is catalyzed by lymphocyte-specific protein tyrosine kinase (Lck) and may influence downstream signaling, as demonstrated by reduced activation of transcription factors when Y90 is mutated to Phe [33,58]. T219, which is conserved in all species and also present in PKCδ, has been reported to be an autophosphorylation site that regulates PKCθ translocation to the membrane [51]. Given the abundance of phosphorylations noted in unbiased phosphoproteomic analyses, future studies are likely to unveil new regulatory mechanisms by this post-translational modification.

## Activation of PKCθ

**Figure 3:**
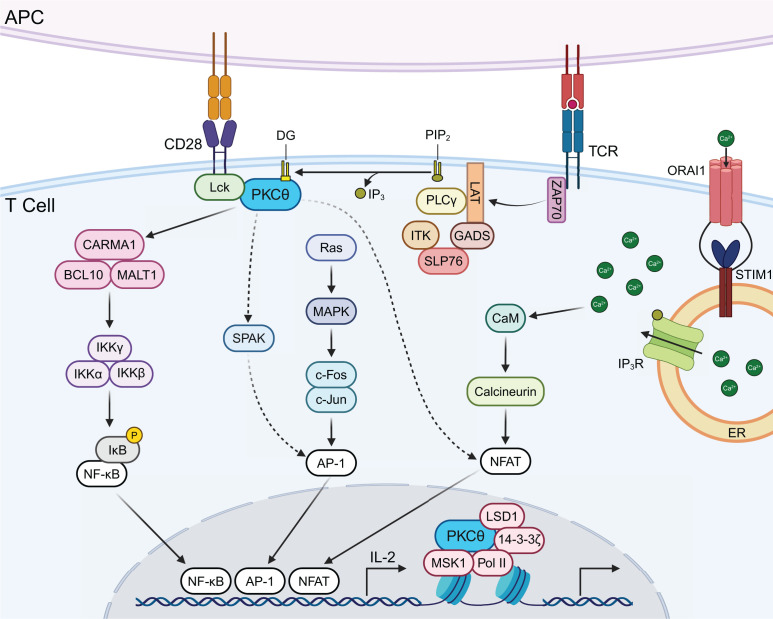
PKCθ signaling in T cells PKCθ activation requires TCR engagement and CD28 co-stimulation. CD28 engagement facilitates the selective recruitment of PKCθ to the immunological synapse, leading to the formation of a trimolecular complex mediated by Lck. TCR engagement leads to PLCγ activation and hydrolysis of PIP_2_ into DG, which binds and activates PKCθ. Once activated, PKCθ initiates downstream signaling leading to NF-κB, AP-1, and NFAT activation and expression of immune response genes, such as IL-2. PKCθ can additionally translocate into the nucleus, where it functions as part of a chromatin-bound transcription complex to regulate gene expression. APC, antigen presenting cell; AP-1, activator protein 1; BCL10, B cell lymphoma/leukemia 10; CaM, calmodulin; CARMA1, caspase recruitment domain-containing membrane-associated guanylate kinase protein-1; DG, diacylglycerol; ER, endoplasmic reticulum; GADS, (GRB2-related adaptor downstream of Shc); IKK, IκB kinase; IκB, inhibitor of κB; IL-2, interleukin-2; IP3, inositol trisphosphate; IP3R, IP3 receptor; ITK, interleukin-2-inducible T cell kinase; LAT, linker for activation of T cells; Lck, lymphocyte-specific protein tyrosine kinase; LSD1, lysine-specific demethylase 1; MALT1, mucosa-associated lymphoid tissue lymphoma translocation 1; MAPK, mitogen-activated protein kinase; MSK1, mitogen- and stress-activated kinase 1; NFAT, nuclear factor of activated T cells; NF-κB, nuclear factor kappa-light-chain-enhancer of activated B cells; ORAI1, ORAI calcium release-activated calcium modulator 1; PIP2, phosphatidylinositol 4,5-bisphosphate; PKCθ, protein kinase C theta; PLCγ, phospholipase C gamma; Pol II, RNA polymerase II; SLP76, SH2 domain-containing leukocyte protein of 76 kDa; SPAK, STE20/SPS1-related proline-alanine-rich kinase; STIM1, stromal interaction molecule 1; TCR, T cell receptor; ZAP70, zeta-chain-associated protein kinase 70.

Signals that cause the hydrolysis of phosphatidylinositol 4,5-bisphosphate (PIP_2_) by phospholipase C (PLC) elevate DG and result in intracellular Ca^2+^ release (Figure 2B) [59,60]. Unlike cPKCs, which require both DG and Ca^2+^, DG alone is sufficient to recruit PKCθ to the plasma membrane [24,61]. Engagement of the C1B domain with DG induces a conformational change that releases the pseudosubstrate from the substrate-binding pocket, enabling downstream signaling (Figure 2B; [3]) [62]. A reduction in DG will return PKC to its autoinhibited state, in which the enzyme is inactive but ready to respond to an increase in DG (Figure 2B, [2]) [63]. If PKC remains in the open and active conformation for too long, as is the case with the tumor-promoting phorbol esters, PKC is susceptible to dephosphorylation at the hydrophobic motif by PH domain leucine-rich repeat protein phosphatase 1 [56,64,65]. This, in turn, triggers dephosphorylation at the turn motif and activation loop by protein phosphatase 1 and 2A [53,66,67]. Following dephosphorylation, PKC is ubiquitinated and degraded by the proteasome in a process known as down-regulation (Figure 2B; [4,5]) [55,56,68]. For PKCθ, mutation of K413 confers resistance to degradation, suggesting a role for this residue in ubiquitin-mediated down-regulation [69]. The E3 ligase Pellino1 (Peli1) has been shown to ubiquitinate PKCθ; however, it remains unclear whether Peli1 targets K413 [69,70].

## Tools to study PKCθ

Although there is no full-length structure of any PKC isozyme, several structures of the kinase domain of PKCθ have been solved in complex with inhibitors. A notable inhibitor is the diaminopyrimidine Compound 20 (C20) discovered in an unbiased high-throughput screen for specific PKCθ inhibitors [71]. C20 exhibits high potency toward PKCθ, with a reported IC_50_ of 0.018 μM [71]. In cellular assays, we showed that 1 μM C20 abolished PKCθ activity, had no effect on cPKCs, aPKCs, or novel-e PKCs, and was significantly less potent towards the other novel-d PKC, PKCδ [11]. Phospho-specific antibodies to the phosphorylated turn motif [72], the activation loop [73], Y90 [74], and T219 [75] provide insight into the phosphorylation state of PKCθ. It is important to note that phosphorylation of the PKCθ turn motif and hydrophobic motif is constitutive and the phosphorylation at these sites is not a readout of enzyme activity; rather, these phospho-specific antibodies monitor steady-state levels of PKCθ. C-kinase activity reporters [76,77] are recognized by PKCθ and, in combination with specific inhibitors such as C20, provide tools to dissect the contributions of PKCθ signaling in cells [11].

## PKCθ signaling in T cells

Most of our understanding of PKCθ signaling comes from studies in T cells. Engagement of the TCR with antigen-presenting cells initiates a signaling cascade that leads to PKCθ activation, which in turn activates downstream transcription factors, driving proliferation and cytokine production (Figure 3) [78]. PKCθ is uniquely recruited to the central supramolecular activation cluster of the immunological synapse through a cooperative interaction between the TCR and CD28 co-receptor [79,80]. This localization requires the Src family kinase Lck, which acts as an adaptor to bridge a phosphorylated tyrosine in the C-terminal proline-rich motif of CD28 to a proline-rich motif in the V3 domain of the PKCθ hinge region [26,81–83]. Mutation of the motif on PKCθ disrupts its recruitment to the immunological synapse and T cell activation, while its insertion into PKCδ is sufficient to confer localization to the immunological synapse [26]. Recently, the peptidyl-prolyl cis-trans isomerase, Pin1, was shown to also associate with the V3 domain of PKCθ and may influence its localization and/or activity [84,85].

Upon recruitment to the immunological synapse, PKCθ is activated by DG produced downstream of PLCγ activation and, once active, drives signaling to NF-κB, AP-1, and NFAT (Figure 3) [8,86]. These transcription factors induce the expression of immune-related genes, such as interleukin-2 (IL-2), which is critical for T cell proliferation and immune response [8]. Despite significant gaps in our understanding of these distinct pathways, studies have shown that all three transcription factors are impaired when PKCθ is knocked out [4,7,87].

## NF-κB activation

Of the three transcription factors, the role of PKCθ is best understood in NF-κB activation. PKCθ directly binds and phosphorylates caspase recruitment domain (CARD)-containing membrane-associated guanylate kinase protein 1 (CARMA1, also known as CARD11), a scaffold that coordinates downstream signaling [88–90]. PKCθ-mediated phosphorylation of CARMA1 within its linker region induces a conformational change that relieves autoinhibition and promotes its oligomerization and membrane association [89]. Activated CARMA1 then recruits B cell lymphoma/leukemia 10 (BCL10) and mucosa-associated lymphoid tissue lymphoma translocation protein 1 (MALT1) to assemble the CARMA1–BCL10–MALT1 (CBM) signalosome (Figure 3) [91]. The CBM complex serves as a signaling hub that recruits the E3 ubiquitin ligase TRAF6, leading to K63-linked polyubiquitination of MALT1 and recruitment of ubiquitin-binding adaptors TAB2 and TAB3 [92,93]. These adaptors facilitate the recruitment of the kinase TAK1 and the regulatory subunit of the IKK complex, IKKγ [93]. TRAF6 ubiquitinates IKKγ, promoting TAK1-mediated phosphorylation and activation of the IKK complex. Activated IKKβ then phosphorylates IκBα, targeting it for proteasomal degradation and thereby releasing NF-κB dimers (p65/p50) to translocate to the nucleus and drive transcription of target genes such as IL-2 [93–95]. Notably, stimulated PKCθ^−/−^ T cells fail to degrade IκB, indicating defective IKK complex activation and establishing a clear link between PKCθ and NF-κB signaling [4]. The formation and function of the CBM complex are not restricted to T cells; in B cells, PKCβ-mediated phosphorylation of CARMA1 drives NF-κB activation following B cell receptor engagement, underscoring the importance of the PKC/CARMA1 signaling axis in immune cell biology [96].

## AP-1 activation

PKCθ is also implicated in AP-1 activation, as PKCθ^−/−^ T cells exhibit impaired AP-1 activity [4]. In T cells, AP-1 activation is mediated by the Ras/MAPK pathway, which may be influenced by PKCθ; however, the precise link remains unclear [97,98]. JNK and ERK, two key members of the MAPK pathway, are possible candidates given their roles in AP-1 activation through phosphorylation of c-Jun and c-Fos, respectively [97,98]. However, studies have yielded conflicting results regarding their dependency on PKCθ. Some reports showed that JNK and ERK activation were unaffected in PKCθ^−/−^ T cells [4,7], whereas others showed that their activation was impaired when PKCθ was knocked out or inactivated [99–102]. Phosphorylation of c-Jun and c-Fos promotes their dimerization, forming the functional AP-1 complex, which appears to be at least partially regulated by PKCθ (Figure 3) [97,98]. A recent study identified STE20-related proline-alanine-rich kinase (SPAK, STLK3), a putative MAP3K, as a direct target of PKCθ [103]. PKCθ-SPAK interaction has been shown to be enhanced upon TCR/CD28 co-stimulation, coinciding with SPAK activation that is disrupted in PKCθ^−/−^ T cells [103]. Moreover, constitutively active PKCθ failed to activate AP-1, but not NF-κB, when co-expressed in T cells with SPAK RNAi or a dominant-negative SPAK mutant [103]. These findings suggest that SPAK may serve as the critical link between PKCθ and AP-1 activation in T cells.

## NFAT activation

The regulation of NFAT activation by PKCθ remains the least understood among its signaling pathways, but evidence suggests a link through Ca^2+^ signaling. NFAT activation is driven by the Ca^2+^/calcineurin pathway, in which PLCγ hydrolyzes PIP_2_ to generate IP_3_, triggering Ca^2+^ release from the endoplasmic reticulum (ER) [104,105]. The resulting depletion of ER Ca^2+^ stores is sensed by the ER-resident protein, stromal interaction molecule 1 (STIM1), which then activates the plasma membrane Ca^2+^ channel, ORAI1, to mediate sustained Ca^2+^ influx [106]. The elevated cytosolic Ca^2+^ binds to calmodulin (CaM), which then activates the phosphatase calcineurin, leading to NFAT dephosphorylation and nuclear translocation (Figure 3) [104,105]. Early studies with T cells from PKCθ knockout mice revealed reduced NFAT activation, which correlated with reduced intracellular Ca^2+^ mobilization [7,87], suggesting that PKCθ controls Ca^2+^ levels to regulate NFAT dephosphorylation/activation. Although the mechanism remains to be established, evidence supports PKCθ activation promoting PLCγ activation via the Tec-family of tyrosine kinases, which directly activate PLCγ [107–110]. Notably, the Tec-family member, Tec, has been shown to constitutively associate with PKCθ, where it enhances signaling downstream of PLCγ1 to provide positive feedback regulation [111]. Consistent with this, Tec activation, PLCγ phosphorylation, and Ca^2+^ influx were reported to be reduced in PKCθ^−/−^ T cells, supporting a role for PKCθ in promoting Ca^2+^ signaling [111]. Tec itself is necessary for NFAT activation, and its overexpression is sufficient to induce PLCγ phosphorylation and NFAT activation in lymphocytes [108]. Another Tec-family kinase, ITK, associates with adaptor proteins at the plasma membrane during T cell activation, and its loss results in defects in Ca^2+^ influx, proliferation, and cytokine production (Figure 3) [107–110]. The current model suggests that TCR/CD28-induced activation of PLCγ generates DG, which in turn activates PKCθ. PKCθ then sustains PLCγ activation via Tec kinases, promoting IP_3_ production and prolonged Ca^2+^ release, ultimately facilitating NFAT activation and T cell responses. However, key questions remain unresolved, including whether PKCθ activity toward a defined Tec kinase or PLCγ intermediate is necessary and sufficient to drive NFAT activation.

## Nuclear role of PKCθ

A nuclear mechanism has also been proposed for PKCθ to regulate T cell gene expression [112–117]. In this model, canonical cytoplasmic activation of PKCθ downstream of TCR signaling is followed by the nuclear translocation of a subset of PKCθ, although the molecular determinants governing this process remain incompletely defined. Upon T cell stimulation, PKCθ translocates to the nucleus, a process proposed to involve interactions with adaptor proteins and post-translational modifications, where it forms an active transcription complex with RNA polymerase II, mitogen- and stress-activated kinase 1 (MSK1), lysine-specific demethylase 1 (LSD1), and adaptor molecule 14-3-3ζ (Figure 3) [115]. This complex associates with regulatory regions of inducible immune response genes, such as CD69, TNF-α, and IFN-γ [115]. In this context, PKCθ may phosphorylate nuclear proteins to modulate their activity or function as a structural adaptor that stabilizes the transcription complex. Additionally, PKCθ has been implicated in the negative regulation of a subset of microRNAs, possibly by binding to their promoter regions, thereby influencing post-transcriptional gene regulation [116]. Notably, most evidence supporting nuclear PKCθ function has been derived from T cells, and whether similar mechanisms operate in other cell types or under distinct signaling contexts remains an open question. Together, these findings reveal PKCθ as a multifaceted regulator of immune signaling, integrating cytoplasmic and nuclear functions to finely tune T cell activation and immune responses.

## Protective role of PKCθ in T cell exhaustion

Recent evidence suggests that PKCθ plays a protective role in T cell exhaustion, a state resulting from chronic T cell stimulation (e.g., in cancer or infection) that leads to diminished immune function [69,70,118]. Experiments using CD8^+^ T cells revealed that chronic stimulation leads to PKCθ degradation and terminal exhaustion, as indicated by an exhaustion-associated gene-expression signature, including loss of TCF1, elevated TIM3 and TOX, and reduced IFNγ [69]. This exhausted state may result, at least in part, from impaired CARMA1 pathway activation caused by PKCθ degradation, which is mediated by the E3 ligase Peli1; accordingly, Peli1-deficient CD8^+^ tumor-infiltrating cells were protected from T cell exhaustion [70]. Supporting a protective role for PKCθ, expression of a degradation-resistant PKCθ mutant improved anti-tumor CD8^+^ T cell responses in mouse models, and human CAR-T cells lacking PKCθ exhibit impaired function *in vitro*, highlighting its translational relevance in immunotherapy [69].

## Non-immune functions of PKCθ

Beyond the immune system, PKCθ is also expressed in skeletal muscle, where it is the most abundant PKC isozyme and has been shown to regulate insulin signaling and glucose metabolism [35,119]. Chronic high-fat diets lead to elevated levels of circulating free fatty acids and the accumulation of DG in skeletal muscle, promoting PKCθ activation [120–122]. Once activated, PKCθ phosphorylates insulin receptor substrate-1 (IRS-1) at Ser1101, inhibiting its tyrosine phosphorylation in response to insulin receptor activation and thereby disrupting downstream signaling through the PI3K/Akt pathway [123]. This impairs key insulin-dependent processes such as glucose uptake and glycogen synthesis, contributing to insulin resistance [124]. Accordingly, PKCθ has been implicated in Type 2 diabetes, with knockout studies in mice demonstrating protection against diet-induced insulin resistance [125].

PKCθ is also highly expressed in platelets, where it plays an important role in hemostasis and thrombus formation [126,127]. It functions downstream of glycoprotein VI (GPVI) and protease-activated receptor (PAR) to phosphorylate Wiskott–Aldrich syndrome protein–interacting protein (WIP), tSNARE protein syntaxin-4, and ERK [128,129]. As such, PKCθ contributes to the regulation of α-granule secretion, platelet adhesion, spreading, and aggregation [130,131]. The potential therapeutic relevance of PKCθ in platelet function remains to be explored.

## PKCθ in disease

### Cancer

Emerging mechanistic and patient survival data are reframing PKC isozymes, including PKCθ, as generally having tumor-suppressive roles. The discovery in the early 1980s that PKC is activated by tumor-promoting phorbol esters led to the assumption that it was an oncogene [132]. This led to the development of cancer therapies that inhibited PKC. In clinical trials, however, these drugs failed to improve patient outcomes, with some worsening the disease [132,133]. A 2015 meta-analysis of five non-small cell lung carcinoma trials revealed a decrease in response rates and disease control in patients receiving chemotherapy in combination with PKC inhibitors compared with chemotherapy alone [133]. We now know that constitutive activation of PKC, which phorbol esters cause, results in its paradoxical inactivation by triggering its dephosphorylation and subsequent degradation [134]; this down-regulation contributes to the tumor-promoting properties of phorbol esters [135]. Moreover, overexpression of the novel-d PKCδ prevents phorbol ester-induced tumorigenesis in mouse models of skin carcinogenesis [136]. PKC was reframed as having primarily tumor-suppressive functions with the finding that cancer-associated mutations spanning all four subfamilies are generally loss-of-function [68].

Mounting evidence supports PKCθ as also having a primarily tumor-suppressive function. Like the many characterized cancer-associated PKC mutations in other family members [68], analysis of cancer-associated mutations in PKCθ has recently revealed that they are also loss-of-function [11]. Specifically, characterization of cancer-associated mutations in PKCθ, including two in the pseudosubstrate, one in the C1A domain, and one in the C-tail, revealed they reduced function. They did so through two distinct mechanisms: mutations that disrupted autoinhibition led to protein instability and rapid degradation, whereas mutations that strengthened autoinhibition resulted in reduced kinase activity [11]. In support of a tumor-suppressive function, bioinformatics analysis of multi-omics data revealed that PKCθ mRNA and protein levels were reduced in lung, colorectal, and pancreatic cancers [11]. Although PKCθ expression is not typically associated with epithelial cells, its detection likely reflects infiltrating immune cells. Consistent with a tumor-suppressive role for PKCθ, the study identifying Peli1 as the E3 ligase regulating PKCθ ubiquitination also reported that Peli1 knockdown prevented T cell exhaustion and enhanced anti-tumor T cell responses [70]. Thus, loss-of-function cancer-associated mutations and reduced expression in diverse tumors suggest PKCθ is generally tumor suppressive.

Other studies of PKCθ in cancer suggest that there may be contexts where it functions as an oncogene [10]. A notable example is gastrointestinal stromal tumors (GIST), where PKCθ is highly expressed, contrasting with other mesenchymal or epithelial tumors where it is absent [137–145]. Cellular studies support an oncogenic function: PKCθ knockdown in GIST cells resulted in reduced cell proliferation, pointing to a potential oncogenic role in these tumors [141].

The role of PKCθ in breast cancer is another example of a context-specific function. In invasive breast cancer, analysis of patient data reveals that high protein expression of PKCθ confers a significant survival advantage (proteinatlas.org), consistent with tumor-suppressive functions [146]. This is not the case for estrogen receptor-negative tumors, where levels of PKCθ are elevated compared with healthy breast tissue [147,148], or for triple-negative breast cancer, where PKCθ inhibition has been shown to reduce tumor growth [149] and improve responses to chemotherapy drugs [150]. Mechanistic studies in MCF-7 and ZR-75 breast cancer cells indicate that PKCθ activates the Akt/FOXO3A pathway, resulting in the inhibition of ERα expression, which leads to c-Rel activation and the up-regulation of genes involved in transformation, such as c-Myc [147]. Additionally, PKCθ has been implicated in metastasis through the activation of FAK [151], as well as by phosphorylating the AP-1 family member Fra-1, leading to the expression of genes associated with migration and invasion [152,153]. Interestingly, the nuclear PKCθ complex may play a significant role in breast cancer by binding and regulating genes involved in epithelial-mesenchymal transition [115,154–156]. In this regard, selective targeting of PKCθ entry into the nucleus may have therapeutic value: a peptide that selectively inhibits the nuclear translocation of PKCθ has shown promise in inhibiting mesenchymal signatures in murine triple-negative breast cancers [117].

Cellular studies continue to unmask the complex role of PKCθ in cancer. In lung cancer, PKCθ appears to play an oncogenic role through activation of TBK1 [157], whereas in renal cancer, it seems to have a protective role [158,159]. Notably, natural compounds such as tonantzitlolone and englerin A have demonstrated a cytotoxic effect on renal cancer cells through the activation of PKCθ [158,159]. In Notch3-dependent T cell lymphoma, PKCθ has been reported to promote T cell survival by activating the NF-κB pathway downstream of Notch3 [160]. In contrast, in leukemia, PKCθ appears to play a protective role, as wild-type mice exposed to the Moloney–murine leukemia virus had a lower incidence of disease compared with PKCθ-deficient mice [161]. Despite the growing body of evidence linking PKCθ to cancer, its precise role remains unclear and may be cancer-type specific. Further studies are needed to explore PKCθ function in cancers that have not yet been investigated. Moreover, the role of the immune system in cancer should also be considered, as PKCθ may influence the disease through immune signaling rather than driving it directly.

### Autoimmune and inflammatory disorders

Many studies investigating the role of PKCθ in autoimmune diseases rely on PKCθ knockout mice, which consistently exhibit reduced disease incidence and severity [162]. In conditions such as arthritis [163], myocarditis [164], hepatitis [165], and colitis [166–168], this reduction is accompanied by decreased T cell infiltration and pro-inflammatory cytokine production compared with wild-type mice [169]. PKCθ has also been implicated in multiple sclerosis (MS), as PKCθ-deficient mice immunized with myelin oligodendrocyte glycoprotein showed a lower incidence of experimental autoimmune encephalomyelitis, a model of MS [170,171]. Additionally, genome-wide association studies have identified potential links between polymorphisms in the *PRKCQ* locus and autoimmune diseases, such as type 1 diabetes [172], rheumatoid arthritis [173–175], celiac disease [174], and Crohn’s disease [176], where specific SNPs are associated with an increased risk for these diseases.

PKCθ has been implicated in the regulation of allergic responses, particularly through activation and differentiation of T helper (Th) cells [177]. In allergic diseases, PKCθ was shown to be essential for the activation of Th2 cells, which produce pro-inflammatory cytokines such as IL-4, IL-5, and IL-13, which drive IgE production, eosinophil recruitment, and airway hyperresponsiveness [178,179]. In asthma models, PKCθ-deficient mice exhibited reduced Th2 responses and decreased inflammation [178,179]. These findings suggest that targeting PKCθ could be a potential strategy for modulating allergic inflammation and related diseases.

PKCθ has also been implicated in graft-versus-host disease (GvHD), a condition in which donor T cells attack host tissues following allogeneic transplantation [180]. In a bone marrow transplantation mouse model, PKCθ was essential for GvHD induction, as PKCθ-deficient T cells protected from GvHD while retaining graft-versus-leukemia effects [180]. Furthermore, PKCθ has been implicated in allograft rejection, in part through its regulation of Bcl-xL, an anti-apoptotic protein that promotes the survival and expansion of alloreactive T cells [181]. In a cardiac transplantation model, PKCθ-deficient mice exhibited increased tolerance to cardiac allografts, demonstrating a role for PKCθ in T cell-mediated rejection [181]. Moreover, regulatory T cells treated with cell-penetrating PKCθ antibodies showed enhanced suppressive activity and, when transferred into mouse models of GvHD, led to reduced disease severity [182]. These findings suggest that targeting PKCθ could improve transplantation outcomes by mitigating GvHD [183].

## Translational significance

Many PKCθ-specific inhibitors have been developed and show promise in research [71,184–189]; however, translating these into clinical use remains challenging due to limited selectivity and potential off-target effects. As noted previously, decades of PKC-targeted inhibitors in cancer trials failed to improve patient outcomes and, in some cases, worsened disease, highlighting the need to better understand PKCθ in order to target it effectively. Although several PKC inhibitors have been evaluated clinically for autoimmune or inflammatory diseases, none are specific to PKCθ [9]. For example, sotrastaurin (AEB071), a pan-PKC inhibitor, has been tested in multiple trials targeting PKCθ. In kidney transplantation, it showed promising effects in combination with tacrolimus (phase II, NCT00403416), but a high rate of acute rejection was observed when combined with mycophenolic acid (phase II, NCT00492869) [190]. In uveal melanoma, combination therapy with alpelisib (phase Ib, NCT02273219) [191] or binimetinib (phase Ib, NCT01801358) [192] yielded no remissions, with only a few patients achieving stable disease. A study in patients with psoriasis reported that AEB071 was well tolerated and reduced symptom severity [193]. The PKCθ-specific inhibitor, CC-90005 [188], entered a phase I clinical trial for psoriasis but was terminated (NCT02502188). In 2023, a PKCθ-specific small-molecule inhibitor (EXS4318) was developed for autoimmune and inflammatory disorders and was approved for a phase I clinical trial; however, it was later withdrawn [194]. To date, no clinical trials involving PKCθ activators have been reported. An important consideration is that inhibiting PKCθ may have unintended consequences in cancer, where loss-of-function mutations, but not gain-of-function mutations, have been identified [11]. Thus, targeting PKCθ will need to be disease- and context-specific, and rather than broad inhibition, focusing on specific downstream pathways may offer greater therapeutic benefits while minimizing off-target effects.

## Conclusion

PKCθ is best known for its role in immune signaling, yet many aspects of its function are incompletely understood. Although it is well-established that PKCθ is essential for T cell activation through its regulation of NF-κB, AP-1, and NFAT, the precise mechanisms linking PKCθ to transcription factor activation remain unclear. Resolving these gaps is crucial, particularly in the context of autoimmune diseases, inflammatory disorders, and cancer, where PKCθ dysregulation has been implicated. Continued research will be critical for fully elucidating the mechanisms that govern this complex enzyme in both health and disease.

## Open Access

Open access for this article was enabled through an open access agreement between the Biochemical Society/Portland Press and the University of California.

## Abbreviations

CARMA1 (CARD11): caspase recruitment domain-containing membrane-associated guanylate kinase protein 1
**CBM**: CARMA1–BCL10–MALT1
C20: Compound 20
DG: diacylglycerol
GvHD: graft-versus-host disease
IL-2: interleukin-2
PIP_2_: phosphatidylinositol 4,5-bisphosphate
PKC: Protein kinase C
PKCθ: PKC theta
PLC: phospholipase C
TCR: T cell receptor
TIM: TOR-interaction motif
